# Functional Characterization of Novel *Faecalibacterium prausnitzii* Strains Isolated from Healthy Volunteers: A Step Forward in the Use of *F. prausnitzii* as a Next-Generation Probiotic

**DOI:** 10.3389/fmicb.2017.01226

**Published:** 2017-06-30

**Authors:** Rebeca Martín, Sylvie Miquel, Leandro Benevides, Chantal Bridonneau, Véronique Robert, Sylvie Hudault, Florian Chain, Olivier Berteau, Vasco Azevedo, Jean M. Chatel, Harry Sokol, Luis G. Bermúdez-Humarán, Muriel Thomas, Philippe Langella

**Affiliations:** ^1^Commensals and Probiotics-Host Interactions Laboratory, Micalis Institute, Institut National de la Recherche Agronomique, AgroParisTech, Université Paris-SaclayJouy-en-Josas, France; ^2^Université Clermont Auvergne, Centre National de la Recherche Scientifique UMR 6023 Laboratoire Microorganismes: Génome et EnvironnementClermont-Ferrand, France; ^3^Department of General Biology, Federal University of Minas GeraisBelo Horizonte, Brazil; ^4^AVENIR Team Gut Microbiota and Immunity Equipe de Recherche Labélisée (ERL), Institut National de la Santé et de la Recherche Médicale U1157/UMR7203, Faculté de Médecine Saint-Antoine, Université Pierre et Marie CurieParis, France; ^5^Service de Gastroentérologie, Hôpital Saint-Antoine, Assistance Publique—Hôpitaux de ParisParis, France

**Keywords:** probiotic, commensal, *Faecalibacterium*, molecular and metabolic characterization, immune-modulatory properties

## Abstract

*Faecalibacterium prausnitzii* is a major member of the Firmicutes phylum and one of the most abundant bacteria in the healthy human microbiota. *F. prausnitzii* depletion has been reported in several intestinal disorders, and more consistently in Crohn's disease (CD) patients. Despite its importance in human health, only few microbiological studies have been performed to isolate novel *F. prausnitzii* strains in order to better understand the biodiversity and physiological diversity of this beneficial commensal species. In this study, we described a protocol to isolate novel *F. prausnitzii* strains from feces of healthy volunteers as well as a deep molecular and metabolic characterization of these isolated strains. These *F. prausnitzii* strains were classified in two phylogroups and three clusters according to 16S rRNA sequences and results support that they would belong to two different genomospecies or genomovars as no genome sequencing has been performed in this work. Differences in enzymes production, antibiotic resistance and immunomodulatory properties were found to be strain-dependent. So far, all *F. prausnitzii* isolates share some characteristic such as (i) the lack of epithelial cells adhesion, plasmids, anti-microbial, and hemolytic activity and (ii) the presence of DNAse activity. Furthermore, Short Chain Fatty Acids (SCFA) production was assessed for the novel isolates as these products influence intestinal homeostasis. Indeed, the butyrate production has been correlated to the capacity to induce IL-10, an anti-inflammatory cytokine, in peripheral blood mononuclear cells (PBMC) but not to the ability to block IL-8 secretion in TNF-α-stimulated HT-29 cells, reinforcing the hypothesis of a complex anti-inflammatory pathway driven by *F. prausnitzii*. Altogether, our results suggest that some *F. prausnitzii* strains could represent good candidates as next-generation probiotic.

## Introduction

Despite a large number of bacteria, archaea, viruses, and unicellular eukaryotes inhabit the human body, only a few bacterial genera (*Bacteroides, Clostridium, Bifidobacterium*, and *Faecalibacterium*) predominate in the human gut microbiome (Schmidt, 2013). Nowadays it is recognized that *Faecalibacterium prausnitzii* represents around 5% from the total fecal microbiota in healthy adults (Hold et al., 2003). Furthermore, this bacterium has been proposed to be a sensor and an actor of the human intestinal health. Indeed, the levels of *F. prausnitzii* have been found to be decreased in patients suffering from intestinal and metabolic disorders such as inflammatory bowel diseases (IBD), irritable bowel syndrome (IBS), colorectal cancer (CRC), obesity, and celiac disease among others (Balamurugan et al., 2008; Sokol et al., 2008; Neish, 2009; De Palma et al., 2010; Furet et al., 2010; Rajilic-Stojanovic et al., 2011) as well as in frail elderly (van Tongeren et al., 2005). Moreover, this species may be a biomarker of choice to assist in Ulcerative colitis (UC) and Crohn's disease (CD) discrimination (Lopez-Siles et al., 2017).

*F. prausnitzii* has been only described in detail recently probably because it is very difficult to grow as it is an Extremely Oxygen Sensitive (EOS) bacterium (Duncan et al., 2002). Similar to other EOS bacteria, little is known about the biology of *F. prausnitzii* despite its relevance in the human gut ecosystem (Miquel et al., 2014). Most of the data referring *F. prausnitzii* are based on metagenomic studies (Miquel et al., 2013), with only few studies with isolated strains and functional approach (Duncan et al., 2002; Ramirez-Farias et al., 2009; Lopez-Siles et al., 2012; Foditsch et al., 2014). This gap between metagenomic and microbiological data is striking for microbiota-derived EOS bacteria. To reduce this gap, it is now essential to increase the knowledge of several commensal bacterial strains in order to better understand the beneficial effect of this species.

Most of the commercial probiotics do not include dominant commensal human isolates. This is a reason why these probiotic strains do not colonize the human gut and their effects persist only during a short period of time (Schmidt, 2013). Nowadays, there is an increasing interest in the use of commensal bacteria as potential probiotic agents. The reasons are multiple and the most evident is that the role of commensal bacteria in homeostatic crosstalk has started to be unraveled in the last decade (Wrzosek et al., 2013). The domestic probiotic market, with a turnover approaching $7 billion in Europe and $1.7 billion in the US in 2013 (Schmidt, 2013), is expected to grow in the next years. However, these next-generation probiotic-commensal candidates must meet the same criteria than the conventional ones. It means that they should (i) be isolated and well-characterized, (ii) achieve safety requirements, such as the acceptable resistance to antibiotics or the lack of lytic and adhesion capacities, and (iii) show beneficial effects on the host before being considerate as a probiotic. In this sense, the Food and Agriculture Organization of the United Nations (FAO) and the European Food Safe Administration (EFSA) have established several guidelines for the correct definition and evaluation of probiotics on food (FAO/WHO, 2002; Pineiro and Stanton, 2007; Binnendijk and Rijkers, 2013). Regarding *F. prausnitzii*, although little is known about its safety, there is a clear potential of this species as a next-generation probiotic. This was already proposed for livestock animals with the isolation and characterization of *F. prausnitzii* strains from stool of calves and piglets (Foditsch et al., 2014) but also for patients with intestinal dysbiosis-associated illness with the development of specific formulation keeping this EOS bacteria alive at ambient air (Khan et al., 2014). Besides, its beneficial anti-inflammatory effect has been only analyzed *in vitro* and *in vivo* with the reference strain *F. prausnitzii* A2-165 (Sokol et al., 2008) and the biofilm forming strain HTF-F (Rossi et al., 2015). As the probiotic properties are usually strain-specific ones (Pineiro and Stanton, 2007), individual studies are required to assess the anti-inflammatory properties of other *F. prausnitzii* isolated strains.

The aim of this work is to isolate a collection of novel *F. prausnitzii* strains from healthy volunteers in order to characterize them as potential probiotic bacteria in accordance with Novel Food regulatory (Miquel et al., 2015a). We have also validated the collection of viable isolated strains by metabolic and safety tests in order to better understand their biology especially in the gastrointestinal tract. Furthermore, the anti-inflammatory properties of all these strains were validated *in vitro* in order to identify the best potential *F. prausnitzii* strain to be used as a next-generation probiotic.

## Materials and methods

### Isolation of novel extremely oxygen sensitive (EOS) strains

A cohort of healthy volunteers was first established (Table 1) to collect freshly emitted fecal samples used as inocula. All volunteers signed informed consent to provide the samples and an agreement of confidentiality. The complete isolation of EOS strain procedure was performed in an anaerobic chamber (N_2_ = 90%, CO_2_ = 5% and H_2_ = 5%). Briefly, fecal samples were homogenized and serial dilutions performed in order to plate dilutions 10^−8^ and 10^−9^ on YBHI [Brain–heart infusion medium supplemented with 0.5% yeast extract (Difco)] agar supplemented with rumen fluid 20%. After 4 days of incubation at 37°C, single colonies were obtained on plates and 96 varied colonies were selected and isolated in duplicate on YHBHI supplemented with rumen fluid 20% agar plate. A group of plates was placed brought out of the anaerobic chamber for 1 h to eliminate EOS strains and after a long period of incubation (usually between 48 h and 4 days), we performed a negative screening. The EOS colonies were further re-isolated and a specific *F. prausnitzii* PCR (primers Fprau07/Fprau02) was done to identify strains of this specie (Table 2). Finally, a 16S rRNA gene sequencing was performed after complete 16S rRNA amplification using primer FP1 to FP5 (Table 2; MWG France). The viable isolates were stocked at −80°C with 16% of glycerol.

**Table 1:** Studied cohort of healthy humans' volunteers and new *F. prausnitzii* strain identified.

**Subject**	**Sex**	**Age (years)**	**Fecal SCFA (mM)**			**CFU/g**	**% EOS**	**Identified *F. prausnitzii* strains**	**Cultivability of the strain**
			**Butyrate**	**Propionate**	**Acetate**				
A	M	81	nd	nd	nd	4.4 × 10^9^	51	CNCM-I4540	Yes
B	F	59	nd	nd	nd	8.7 × 10^9^	30.7	X	
C	M	54	nd	nd	nd	8.0 × 10^9^	67.7	CNCM-I4541	Yes
								CNCM-I4542	Yes
								S3C12	No
								S3G1	No
D	M	54	21.7	14.8	65.4	8.0 × 10^9^	69	X	
E	F	60	nd	nd	nd	3.5 × 10^10^	40	X	
F	M	53	3.7	4.4	11.6	3.0 × 10^9^	35.4	X	
G	F	26	3	3.9	14	2.0 × 10^9^	37.5	X	
H	F	56	15.8	10.2	27.6	4.8 × 10^9^	56.2	CNCM-I4574	Yes
								CNCM-I4543	Yes
I	M	59	10.1	9.5	27.3	7.8 × 10^9^	33	S9G3	No
								S9D8	No
J	M	34	9.9	12.3	26.8	7.7 × 10^9^	30.7	CNCM-I4644	Yes
								CNCM-I4544	Yes
								S10H3	No
K	F	60	1.1	2	5.3	2.0 × 10^9^	28.1	X	
L	M	40	1.7	2	6.9	7.4 × 10^9^	29.6	CNCM-I4575	Yes
								CNCM-I4573	Yes
								S13A12	No
								S13E3	No
M	F	51	2.5	3.1	10.8	4.6 × 10^9^	53.1	CNCM-I4546	Yes

*All the isolates were obtained from human fecal samples of healthy volunteers consuming omnivorous diets. F, female; M, male; nd, not determined, X, no identified F. prausnitzii strain*.

**Table 2:** Oligonucleotides used in this study and PCR product sizes.

**Primer**	**Oligonucleotide sequence (5′–3′)**	**PCR product size (bp)**	**Use**	**References**
Fprau07	CCATGAATTGCCTTCAAAACTGTT	141	PCR *F. prausnitzii* specific	Sokol et al., 2008
Fprau02	GAGCCTCAGCGTCAGTTGGT			
FP1	AGAGTTTGATCCTGGCTCAG	1,474	16S rRNA complete sequence amplification and sequencing	This study
FP2	ACGGCTACCTTGTTACGACTT			
FP3	GTTGCGGGACTTAACCCAACATC		16S rRNA sequencing	This study
FP4	GTTTTTCTTGAGTAGTGCAGAGG		16S rRNA sequencing	This study
FP5	GATGTTGGGTTAAGTCCCGCAAC		16S rRNA sequencing	This study

### Bacterial strains, cell culture, and growth conditions

The reference strains A2-165 (DSM 17677; Duncan et al., 2002), L2/6 (Barcenilla et al., 2000) and M21/2 (Louis et al., 2004) and the *F. prausnitzii* isolated strains (Table 1) were grown at 37°C in YBHI medium supplemented with cellobiose (1 mg/ml; Sigma), maltose (1 mg/ml; Sigma), and cysteine (0.5 mg/ml; Sigma) in an anaerobic chamber filled with N_2_ = 90%, CO_2_ = 5% and H_2_ = 5%.

HT-29 (ATCC HTB-38) (LGC-Standars) cell line was grown in Dulbecco's Modified Eagle's minimal essential medium (DMEM) (Sigma-Aldrich) supplemented with 10% (w/v) heat-inactivated fetal bovine serum (FBS) (GibcoBRL, Eragny, France) and with penicillin G/ streptomycin (5,000 IU/mL, 5,000 μg/mL) (Sigma-Aldrich). Cultures were incubated in 25 cm^2^ tissue culture flasks (Nunc, Roskilde, Denmark) at 37°C in a 5% (v/v) CO_2_ atmosphere until confluence.

### 16S rRNA gene analysis

DNA was extracted from isolated colonies of the different *F. prausnitzii* strains by alkaline lysis in 50 μL of NaOH 0.5 M during 30 min and 50 μL of Tris 1M pH7 and 100 μL H_2_O were added. 16S rRNA sequences were amplified using FP1 and FP2 primers (Table 2) and PCR products purified with the Wizard SV Gel. PCR Clean-Up system (Promega) was used to obtain bidirectional partial 16S rRNA gene sequences by using primers FP1, FP2, FP3, FP4, and FP5 (Table 2). All DNA sequences were confirmed by sequencing (Eurofins MWG Operon, Ebersberg, Germany). Sequences for the novel isolates were deposited in the NCBI database under the accession numbers MF185398 to MF186168.

Phylogenetic analysis based on 16S rRNA were performed using the multiple sequence alignment—CLUSTALW (Thompson et al., 1994) integrated in MEGA6 software (Tamura et al., 2013). After that, the most appropriate evolutionary model was defined and the evolutionary history was inferred using the Maximum likelihood (ML) criterion, based on the Kimura 2-parameter model (Kimura, 1980), with 1,000 bootstrap replicates. A discrete Gamma distribution was used to model evolutionary rate differences among sites [five categories (+G, parameter = 0.1846)]. The rate variation model allowed for some sites to be evolutionarily invariable ([+I], 64.70% sites). Initial tree(s) for the heuristic search were obtained by applying the Neighbor-Joining method to a matrix of pairwise distances estimated using the Maximum Composite Likelihood (MCL) approach and all positions containing gaps and missing data were eliminated. The tree with the highest log likelihood (−3073.67) is shown (**Figure 3**). The tree is drawn to scale, with branch lengths measured in the number of substitutions per site. The analysis involved 36 nucleotide sequences. There were a total of 1090 positions in the final dataset. In this analysis, sequences used by Lopez-Siles et al. (Duncan et al., 2002; Ramirez-Farias et al., 2009; Lopez-Siles et al., 2012) were included with the objective of compare the new strains to the two phylogroups proposed by that study. *Eubacterium desmolans* was used to root the tree.

### Plasmid presence

The presence of plasmids in the isolated strains were determined following Wizard® Plus SV Minipreps DNA Purification System (Promega) with modifications to adapt it for use with Gram positive bacteria. Briefly, an extra lysis step was performed after centrifugation of liquid overnight (ON) cultures by incubation for 1 h at 37°C with lysozyme (Sigma; 10 mg/ml) in the cell resuspension solution.

### Scanning electron microscopy

Scanning electron microscopy analyses were performed on the MIMA2 platform (INRA, France) with pure pellet of bacterial culture suspended and fixed in 200 μL of glutaraldehyde and 3% ruthenium red during 2 h in an anaerobic chamber and stored at 4°C. Scanning electron microscopy was performed as previously reported (Joly et al., 2010).

### Determination of antibiotics resistance

The minimum inhibitory concentrations (MIC) for 13 antibiotics (including tetracycline, kanamycin, chloranphenicol, linezolid, nupri/dalfopri, trimethoprim, gentamicin, erythromycin, cefpirome, clindamycin, streptomycin, vanomycin, and ampicillin) were determined on Wilkins-Chalgren agar (Difco) according to the E-test procedure, in accordance with the conditions recommended by the supplier (Biomerieux, France). The results were recorded after 48 h of incubation.

### Anti-bacterial assays

The anti-bacterial effect of *F. prausnitzii* supernatants were investigated *in vitro* using the bacteriocin activity assay as previously described (Ramirez-Farias et al., 2009). This anti-bacterial effect was tested on six different bacterial species: three aerobic bacteria (*E. coli* Nissle 1917, *E. coli* DH10B, and *Listeria monocytogenes* 11765), one facultative anaerobic bacterium (*Lactococcus* subsp *cremoris* MG1363), and two obligate anaerobic bacteria (*Clostridium perfringens* ATCC13124 and *Bifidobacterium infantis* DSM20088/ATCC15697). YBHI liquid medium alone was used as negative control.

### Metabolic activities

To determine the metabolic activities of the cultivable strains, API-20A galleries and the gelatin degradation test of API-20E galleries were used according to manufacturer's instructions. For detection of DNase and hemolytic activity, the strains were grown ON and then plated into Methyl green-DNA agar plates (Difco) or blood agar plates (Biomérieux) respectively. The results were recorded after 48 h of incubation. The capacity to grow in presence of mucin was assayed using a defined medium (KH_2_PO_4_: 5.236 g/L, (NH_4_)_2_SO_4_: 4 g/L, NaCl: 4 g/L, CaCl_2_: 30 mg/L, MgCl_2_: 300 mg/L, MnCl_2_: 30 mg/L, FeCl_2_: 8 mg/L, Vitamin B12: 5 mg/L, Vitamin B1: 1 mg/L, Biotin: 1 mg/L, PABA: 1 mg/L, Folic acid: 1 mg/L, Vitamin K: 2 mg/L, cystein 0.5 mg/mL) supplemented with 1.5% mucin (Type II, Sigma-Aldrich).

### Short chain fatty acid (SCFA) analysis

Supernatant concentrations of propionate, acetate, and butyrate were analyzed using gas liquid chromatography (Nelson 1020, Perkin-Elmer, St Quentin en Yvelines, France) as previously described (Lan et al., 2008). Overnight culture (20 h) of *F. prausnitzii* strains were used and culture media as negative control; each measurement for performed at least in triplicate except for fecal samples. SCFA concentrations are expressed in mM.

### Dosage of D- and L-lactate

D- and L-lactate was measured in supernatant of bacterial cultures. This supernatant was precipitated with trichloroacetic acid (10%) and centrifuged at 4,500 g for 20 min at 4°C. Lactate was then measured in the supernatants with the Biosentec D/L lactic acid enzymatic kits according to the manufacturer instructions (Biosentec, Toulouse, France). Overnight culture (20 h) of *F. prausnitzii* strains were used and culture media as negative control; each measurement was performed at least in triplicate.

### Adhesion assays

Monolayers of HT-29 cells were seeded in 24-well tissue culture plates (Nunc) with 1.83 × 10^5^ HT-29 cells/well and cultivated until confluence, culture medium was changed daily. Monolayers were then infected in 1 ml of the cell culture medium without antibiotics and with heat-inactivated FBS at a multiplicity of infection (MOI) of 100 bacteria per epithelial cell. After, 3 h of incubation at 37°C in anaerobic conditions (as describe above), monolayers were washed three times in phosphate-buffered saline (PBS; pH 7.2). The epithelial cells were then lysed with 1% Triton X-100 (Sigma Chemical Company, St Louis, Mo.) in water. Samples were plated onto YHBHI supplemented agar plates to determine the number of CFU corresponding to the total number of cell-associated bacteria. Adhesion to mucin has been performed as previously described by Radziwill-Bienkowska et al. (2014, 2016) Briefly, after an overnight coating of 96 plates (Nunc) with a solution of 10 mg/ml of mucin [Type III mucin from porcine stomach (lyophilized powder, Sigma-Aldrich)] a bacterial suspension (OD_600nm_ = 1) in PBS of each strain was incubated 3-h at 37°C in the anaerobic chamber. Bound cells were stained with crystal violet. Stained bacteria were suspended in 96% ethanol and optical density was determined at 583 nm. All the experiments were performed in triplicate. The adhesion values have been normalized using *Lactobacillus rhamnosus* GG (LGG) a positive control know by their good adhesion properties to mucin (Martin et al., 2015). Results are presented by the mean and the standard deviation.

### Immuno-modulatory properties using HT-29 cells

Anti-inflammatory assays were done following the procedure described by Kechaou et al. (2012). Briefly, 50,000 HT-29 cells per well were seeded in 24-well culture plates (Nunc). Twenty-four h before bacterial co-culture (day 6), the culture medium was changed for a medium with 5% heat-inactivated FBS and 1% glutamine. On the day of co-culture, 10% of bacterial supernatant or bacterial medium (YBHI) were added in DMEM in a total volume of 500 μL. Cells were stimulated simultaneously with human TNF-α (5 ng/ml; Peprotech, NJ) for 6 h at 37°C in 10% CO_2_. All samples were analyzed in triplicate. After co-incubation, cell supernatants were collected and stocked at −80°C until further analysis of interleukin-8 (IL-8) concentrations by ELISA (Biolegend, San Diego, CA). Total protein was determined by Bradford Reagent test (Sigma-Aldrich). Experiments have been done at least in triplicate. Results are expressed as IL-8/protein (pg/mg) and have been normalized using as reference value the IL-8 produced after the co-incubation with PBS as a negative control.

### Experiments on peripheral blood mononuclear cells (PBMCs)

The protocol used in this study was adapted from Kechaou et al. (2012). Commercial PBMCs (StemCell Technologies, France) from five healthy donors were used in this assay. Donors presented the following characteristics: men, age under 65, body mass index <30, non-smoking, no drugs with anti-inflammatory known effects taken during the 15 days prior to sampling, and tested negative for HIV, hepatitis A and B viruses. After reception, cells were stored in liquid nitrogen until use. To prepare PBMCs for co-culture experiments with bacteria, the vial were thawed at 37°C in a water bath and then transferred into a medium containing RPMI-1640 medium supplemented with 10% heat-inactivated FCS, 1% L-glutamine and 0.1% Penicillin/Streptavidin (medium components were bought from Lonza, Switzerland). DNase (100 μg/mL, Roche Applied Science, France) was added to this mix to avoid cell clumping. Cells were then centrifuged at 200 g for 15 min, counted using trypan blue and spread on 24-well plates at 1 × 10^6^ cells/well. Supernatants were added in triplicates (three wells per donor) at 10% in a total volume of 1 ml. Plates were incubated for 24 h at 37°C with 10% CO_2_. Culture supernatant were collected, mixed with an antiprotease cocktail according to manufacturer's instructions (Complete EDTA-Free protease inhibitor, Roche Applied Bioscience) and stored at −80°C until further analysis of IL-10 concentrations by ELISA (Mabtech, Sweden).

### Statistical analysis

GraphPad software (GraphPad Sofware, La Jolla, CA, USA) was used for statistical analysis. Results are presented as bar graphs ±SEM. Comparisons were realized with the non-parametric Kruskal–Wallis test followed by a Dunn's Multiple Comparison test. Correlation test were performed using spearman test. A *p* < 0.05 was considered significant.

## Results and discussion

### Construction of EOS and *F. prausnitzii* libraries

**Figure 1:**
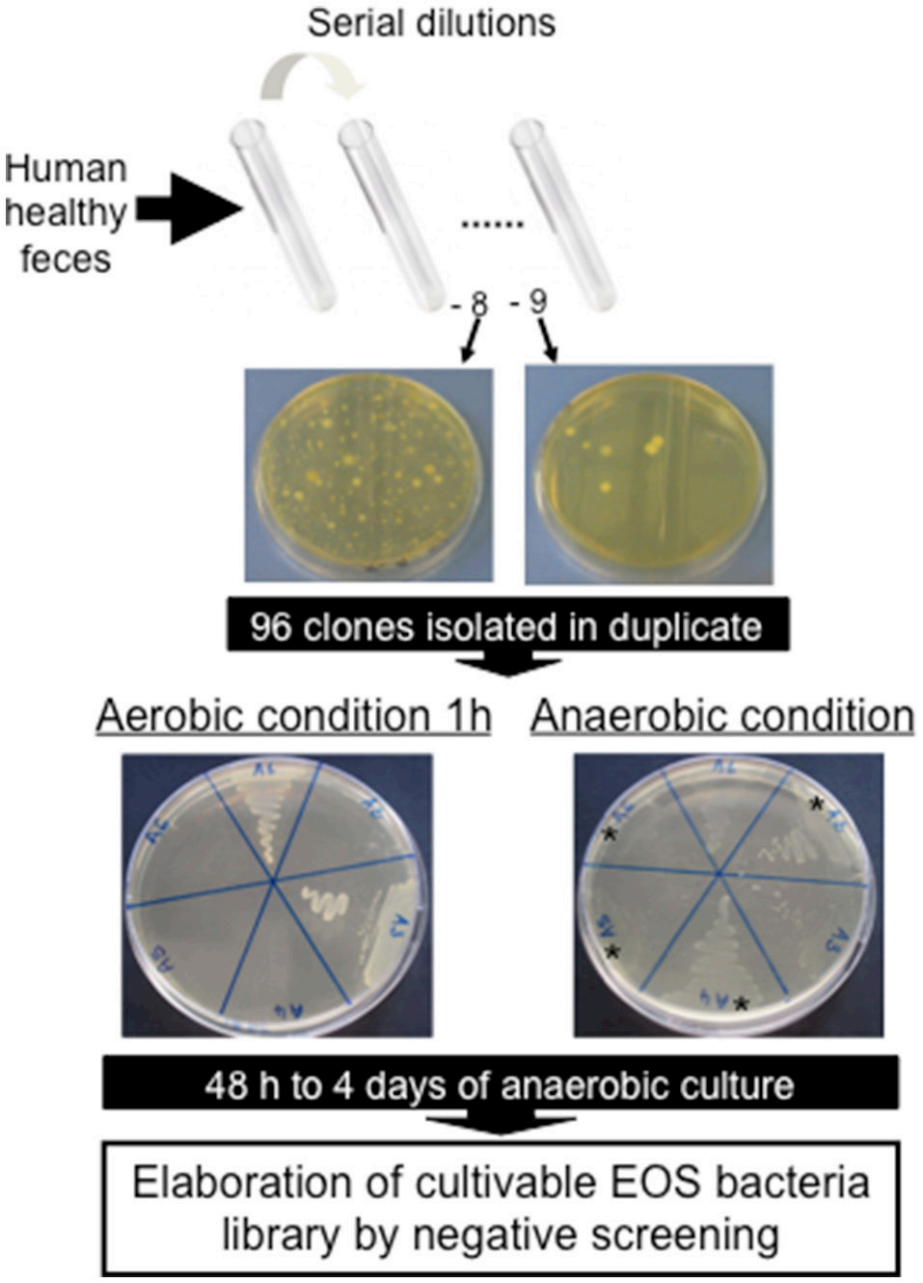
Negative screening for isolation of new Extremely Oxygen Sensitive (EOS) strains from human healthy feces.

**Figure 2:**
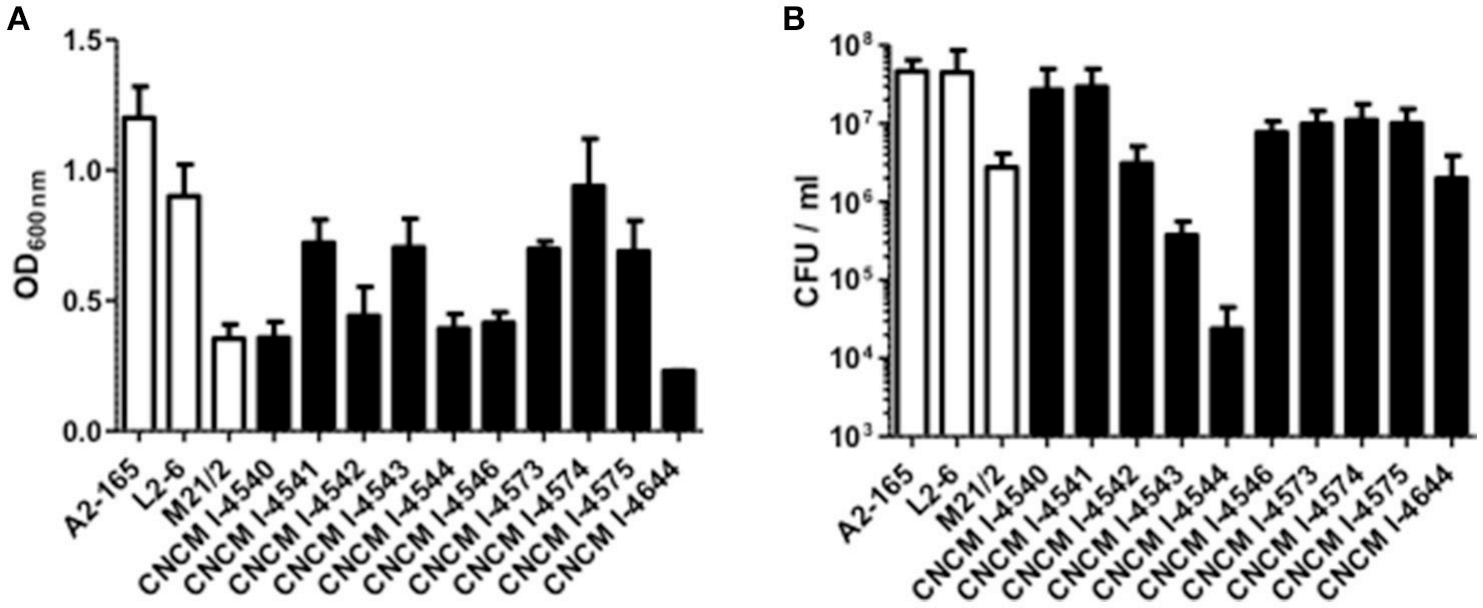
Growth profile of *F. prausnitzii* strains. **(A)** OD_600nm_ determination after 20 h growth in YBHI supplemented medium and **(B)** determination of viable bacteria: the CFU/mL numeration in the same cultures. Each measurement have been done at least in triplicate.

The vast majority of intestinal bacteria are EOS and thus mostly very difficult to culture (Qin et al., 2010). Although metagenomic approaches recently allow identifying some uncultivable organisms, the use of cultivable strains is requested to determine their biological activities. In this study, we report a method for isolation of novel EOS strains from human fecal samples on a complete medium (Figure 1). For this, a negative screening was performed through the exposition of bacterial isolates to oxygen and in parallel, these same strains were cultivated in an anaerobic chamber, which maintains a consistent anaerobic environment to ensure proper conditions for optimal EOS growth. We identified between 28.1 and 67.7% of EOS strains in the microbiota of healthy volunteers (Table 1). Interestingly, the proportion of EOS strains in the human microbiota was positively and significantly correlated to the amount of fecal acetate (*r* = 0.7; *p* = 0.0433) and tend to be correlated to the amount of fecal butyrate (*r* = 0.6833; *p* = 0.0503). These observations suggest that EOS population has an important metabolic impact that could participate to intestinal homeostasis (Wrzosek et al., 2013). The EOS isolates were identified by 16S rRNA gene sequencing and among them *F. prausnitzii* candidate strains were selected for further characterization. These isolation and screening set up can have a small inspecificity rate and no-*F. prausnitzii* strains can be recovered as well as the strain S13E3. After three subcultures, cultivable strains were stored at −80°C in 16% glycerol. Among 17 identified *F. prausnitzii* strains, only 10 were cultivable in the tested conditions (Table 1) with an OD_600 nm_ lower than 2 corresponding to >1 × 10^8^ CFU/mL (Figure 2). There was no direct correlation between CFU counts and OD_600nm_ due to difference of viability between strains. We substantially increased the number of cultured *F. prausnitzii* isolates from human origin and provided new tools for a better understanding of the diversity and microbial ecology of the colon.

### Phylogenetic diversity of *faecalibacterium prausnitzii*

**Figure 3:**
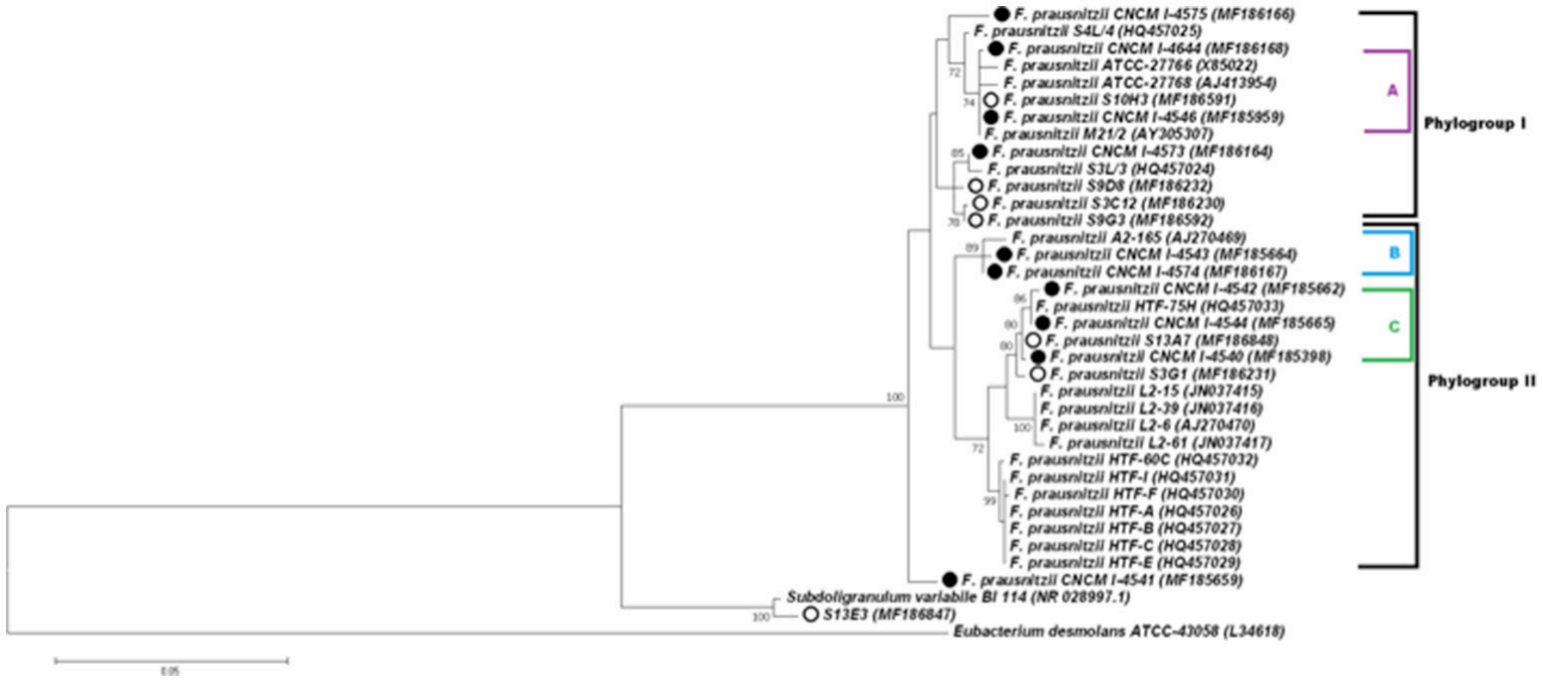
Phylogenetic tree of *F. prausnitzii* strains based on 16S rRNA gene sequences. The tree was constructed with the MEGA6 software package using the Maximum Likelihood method. The bootstrap values above 70% are shown next to the branches. The *F. prausnitzii* isolates incorporated in this study have circles besides. The black circles represent the cultured strains and white circles represent uncultured isolates. Colors (purple, blue, and green) and letters (A, B, and C) indicate the tree groups with high bootstrap values, formed by our cultured strains.

**Figure 4:**
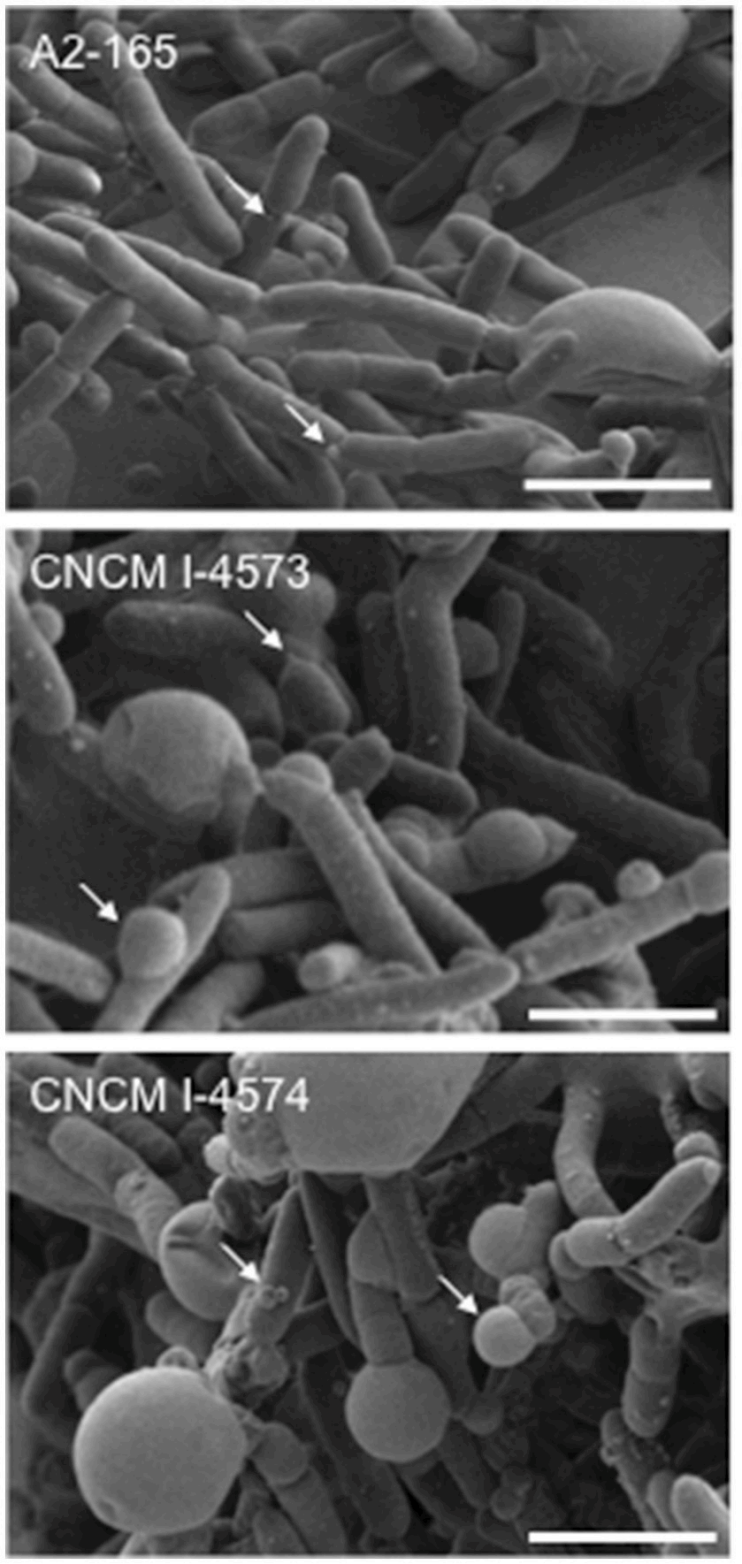
Scanning electron microscopy images of *F. prausnitzii* strains phylogroup II. Strains were grown in YBHI liquid medium 20 h. Scale bars indicate 2 μm. Arrows indicates “swelling.”

Full-length 16S rRNA gene sequences were determined for the 17 isolates of *F. prausnitzii* from healthy individuals (Table 1). The sequences from the literature (Barcenilla et al., 2000; Duncan et al., 2002; FEEDAP, 2012; Lopez-Siles et al., 2012) were included in order to classify the new isolates in the two phylogroups proposed by Lopez-Siles et al. (Barcenilla et al., 2000; Duncan et al., 2002; FEEDAP, 2012; Lopez-Siles et al., 2012; Figure 3). Each of these 16S rRNA sequences were unique, came from a different colony, and share >97% 16S rRNA sequences similarity. Cultivability of strains was not linked to phylogroups affiliation (Figure 3). Of note, all strains have a similar morphotype with cell wall extensions, like “swellings” (Figure 4) already described but with yet unknown function (Miquel et al., 2013). The average nucleotide identity between strains of the two phylogroups (S3L/3 and L2/6 = 94%) supports the hypothesis of the existence of two genomospecies without phenotypic properties defined yet (Lopez-Siles et al., 2017). Although, as was previously described for another library, there was a tendency for some sequences to group by isolation and individual with a clustering of strains (subgroup B of the phylogroup II; Lopez-Siles et al., 2012). For example, CNCM I-4574 and CNCM I-4543 strains were isolated from the same volunteer and present 99.8% of homology at 16S rRNA level.

Interestingly, the existence of strains that do not fit in any phylogroup (as CNCM I-4541) suggest that biodiversity of *F. prausnitzii* remains poorly known, maybe since only few strains have been isolated. Moreover, the strain S13E3, could be not an *F. prausnitzii* stain.

### Resistance to antibiotics

The MIC for the different antibiotics tested are represented in the Table 3. Concerning the breakpoints for Gram positive bacteria from EFSA (Duncan et al., 2004) which classify bacteria as resistant or not to a specific antibiotic, all *F. prausnitzii* isolates were susceptible to clindamycin, vancomycin, ampicillin, quinupristin+dalfopristin, and chloramphenicol (MICs lower than 0.25, 2, 1, 0.5, and 2 mg/L respectively). Only one isolate, the CNCM I-4541 strain was resistant to erythromycin (MICs > 0.5mg/L). Surprisingly, all tested strains were resistant to streptomycin (MICs ranging from 14 to 50 mg/L) excepted for the CNCM I-4575 isolate. Regarding gentamicin, kanamycin, and tetracycline, different results were obtained for the different isolates: with up to 5 isolates displaying resistance to higher concentrations of the tested antibiotics than the determined breakpoint. Finally, three antibiotics (not included in the EFSA guidance) were also analyzed due to their importance in the clinical treatments: trimetroprim, linezolid, and cefpirome. All strains were resistant to trimethoprim, as expected for an anaerobic bacteria (MICs >32 mg/L; data not shown), while they tended to be susceptible to linezolid (MICs ranging from 0.032 to 3.3 mg/L) and resistant to cefpirome (from 4.66 to >256 mg/L) which, when linked to the general susceptibility to ampicillin, might indicate that the penicillin binding proteins of *Faecalibacterium* are poorly recognized by cephalosporins. Remarkably, CNCM I-4543 and CNCM I-4574 isolates were resistant to cefpirome, a fourth-generation cephalosporin stable against most plasmid- and chromosome-mediated beta-lactamases (Wiseman et al., 1997), with a MIC higher than 256 mg/L.

**Table 3:** Minimum inhibitory concentrations (MIC) (mg/L) for the different antibiotics tested.

**EFSA Breakpoint (other Gram +)**	**GEN**	**STR**	**KM**	**ERY**	**CLI**	**VAN**	**TET**	**QD**	**CM**	**AMP**	**CPO**	**LZD**
	**4**	**8**	**16**	**0.5**	**0.25**	**2**	**2**	**0.5**	**2**	**1**	**nd**	**nd**
A2-165	1.37 ± 0.12	**96** ± **18.47**	**8.67** ± **1.76**	0.20 ± 0.08	0.016 ± 0	0.27 ± 0.05	0.016 ± 0	0.03 ± 0.01	0.08 ± 0.02	0.11 ± 0.02	9 ± 1	1.25 ± 0.25
L2-6	**4.33** ± **0.88**	**32** ± **7.15**	0.42 ± 0.08	0.10 ± 0.02	0.023 ± 0	0.47 ± 0.12	**4.21** ± **0.62**	0.58 ± 0.46	**20** ± **5.66**	0.06 ± 0.03	24 ± 4.62	0.62 ± 0.12
M21/2	**4.75** ± **1.49**	**21** ± **4.43**	**51** ± **45**	0.05 ± 0.01	0.016 ± 0	0.25 ± 0	1.09 ± 0.63	0.016 ± 0	0.15 ± 0.03	0.04 ± 0.03	16.67 ± 7.86	0.75 ± 0
CNCM I-4540	1.25 ± 0.25	**21.33** ± **2.67**	**122.67** ± **66.83**	0.11 ± 0.02	0.016 ± 0	0.71 ± 0.18	0.016 ± 0	0.011 ± 0.004	0.05 ± 0.04	0.11 ± 0.02	24 ± 4.62	0.04 ± 0.06
CNCM I-4541	1.62 ± 0.47	**24** ± **0**	**14** ± **2**	**20** ± **4**	0.016 ± 0	0.125 ± 0	**8** ± **0**	0.27 ± 0.24	0.17 ± 0.07	0.06 ± 0	40 ± 8	1.25 ± 0.25
CNCM I-4542	2.87 ± 1.12	**23.67** ± **3.26**	**20** ± **4**	0.11 ± 0.07	0.016 ± 0	0.67 ± 0.17	0.016 ± 0	0.016 ± 0	0.29 ± 0.40	0.08 ± 0.03	20.67 ± 7.69	3.17 ± 1.42
CNCM I-4543	2 ± 0.40	**16** ± **0**	**20** ± **4**	0.08 ± 0.02	0.016 ± 0	0.29 ± 0.04	0.2 ± 0.002	0.07 ± 0.03	0.31 ± 0.09	0.12 ± 0	≥**256** ± **0**	1.25 ± 0.38
CNCM I-4544	**6** ± **1.15**	**21.2** ± **7.31**	**100** ± **52.41**	0.12 ± 0	0.016 ± 0	0.92 ± 0.08	0.016 ± 0	0.023 ± 0	0.11 ± 0.02	0.09 ± 0.02	53.33 ± 5.33	0.5 ± 0
CNCM I-4546	**10** ± **66**	**50.67** ± **13.33**	**256** ± **0**	0.22 ± 0.61	0.018 ± 0.002	0.56 ± 0.31	**2.6** ± **0.6**	0.16+0.07	1.25 ± 1.51	0.07 ± 0.02	24.8 ± 6.24	3 ± 1
CNCM I-4573	**7** ± **1**	**32** ± **0**	**234** ± **21.33**	0.01 ± 0.03	0.024 ± 0.007	0.28 ± 0.05	0.028 ± 0.003	0.04 ± 0.005	0.38 ± 0	0.25 ± 0	22 ± 3.83	3.3 ± 0.8
CNCM I-4574	1.75 ± 0.25	**14** ± **0**	6 ± 2	0.07 ± 0.02	0.016 ± 0	0.25 ± 0	0.016 ± 0	0.03 ± 0.01	0.09 ± 0.02	0.22 ± 0.03	≥**256** ± **0**	0.5 ± 0.14
CNCM I-4575	1.25 ± 0.25	5 ± 1	4 ± 1	0.07 ± 0.01	0.016 ± 0	0.5 ± 0	0.032 ± 0	0.03 ± 0.03	0.016 ± 0	0.084 ± 0.02	4.67 ± 1.67	0.03 ± 0.01
CNCM I-4644	0.91 ± 0.08	**9.33** ± **1.33**	**135** ± **69.84**	0.10 ± 0.05	0.026 ± 0.01	0.23 ± 0.02	0.83 ± 0.32	0.04 ± 0.01	0.079 ± 0.04	0.026 ± 0.01	9.333 ± 1.33	0.58 ± 0.08

*Gentamicin (GEN), streptomycin (STR), kanamycin (KM), erythromycin (ERY), clindamycin (CLI), vancomycin (VAN), tetracycline (TET), quinupristin/dalfopristin (QD), chloramphenicol (CM), ampicillin (AMP), cefpirome (CPO), and linezolid (LZD). Experiments have been done in triplicate and the results are expressed as the media ± SEM. nd, Not defined. In bold, Resistances*.

The analysis of antimicrobial resistance is of major importance due to the fast evolution of antibiotic resistance in response to the extensive use of antimicrobials. However, the microbiological breakpoints marked by the EFSA for most of Gram positive bacteria is probably not the most correct for the analysis of *F. prausnitzii* isolates as no so many information about their natural or acquired resistance patters is reported, to our knowledge, up to day in the literature. Foditsch et al. (2014) have identify that more of the 50% of the *F. prausnitzii* strains that they isolated from fecal samples of healthy calves and piglets were resistant to tetracycline, amikacin, cefepime, and cefoxitin comparing the MIC values with the standard values determined by CLSI for *Bacteroides fragilis* ATCC 25285. This fact highlights the need of more microbiological studies of antibiotic resistance in this species in order to determine a correct standard values for *Faecalibacterium* as well as the search for genes codifying for the most important resistance mechanisms for, at least, some of the antibiotics tested in this study.

### Metabolic activities

Enzymatic activities detected by API-20A gallery system are reported in Table 4. Interestingly, only one enzyme was detected and active in all the tested strains: the beta-galactosidase. Otherwise, all the strains were not able to ferment mannose or raffinose, to reduce nitrate and to produce indole (data not shown). Furthermore, all the isolates were negative for the presence of urease, arginine dihydrolase, beta-glucosidase, alpha-arabinosidase, N-acetyl-beta-glucosaminidase, glutamic acid decarboxylase, alkaline phosphatase, phenylalanine arylamidase, leucine arylamidase, pyroglutamic acid arylamidase, tyrosin arylamidase, alanine arylamidase, glutamyl glutamic acid arylamidase, and serin arylamidase (data not shown). These results confirm previous observations where no strain was able to metabolize arabinose and raffinose among others as the sole energy source (Duncan et al., 2002; Lopez-Siles et al., 2012).

**Table 4:** Metabolic capacities of *F. prausnitzii* strains detected by API 32A galleries.

	**bGal**	**bGP**	**αGLU**	**bGUR**	**ArgA**	**Lga**	**GlyA**	**HisA**
	**beta−galactosidase**	**beta Galactosidase 6 phosphate**	**Alpha glucosidase**	**beta glucuronidase**	**Arginine Arylamidase**	**Leucyl Glycine Arylamidase**	**Glycine Arylamidaseycine**	**Histidine Arylamidase**
A2−165	+	+	+	+	+	+	+	+
L2−6	+	−	+	+	+	+	+	+
M21/2	+	−	−	−	+	−	+	+
CNCM I−4540	+	+	−	−	−	−	−	−
CNCM I−4541	+	+	−	−	+	−	−	−
CNCM I−4542	+	+	−	−	−	−	−	−
CNCM I−4543	+	+	−	+	+	+	+	−
CNCM I−4544	+	−	−	−	−	−	−	−
CNCM I−4546	+	−	−	−	+	−	+	+
CNCM I−4573	+	−	−	−	+	−	+	+
CNCM I−4574	+	+	−	+	+	+	+	−
CNCM I−4575	+	+	−	+	+	−	+	+
CNCM I−4644	+	−	−	−	+	+	+	+

*+, Presence; −, absence. Experiments have been done in triplicate*.

For all the other enzymes (6 phospho-beta galactosidase, alpha-glucosidase, beta-glucuronidase, arginine arylamiase, leucyl glycerine-arylamidase, glycine-arylamidaseycine, and histidine-arylamidase), differences inter-strains were detected (Table 4). Beta-glucuronidase activity has been previously reported in some *F. prausnitzii* isolates (Lopez-Siles et al., 2012). While six strains showed individual profiles, the other seven are included in three different profiles. Two of them corresponds to the group A from phylogroup I (CNCM I-4546 and M21/2). The strains CNCM I-4543 and CNCM I-4574 (group B, phylogroup II), which are the only ones resistant to cefpirome, share also the same metabolic profile and donor. And the third metabolic profile is shared by strains CNCM I-4540 and CNCM I-4542 that belong to the group C of phylogroup II.

**Figure 5:**
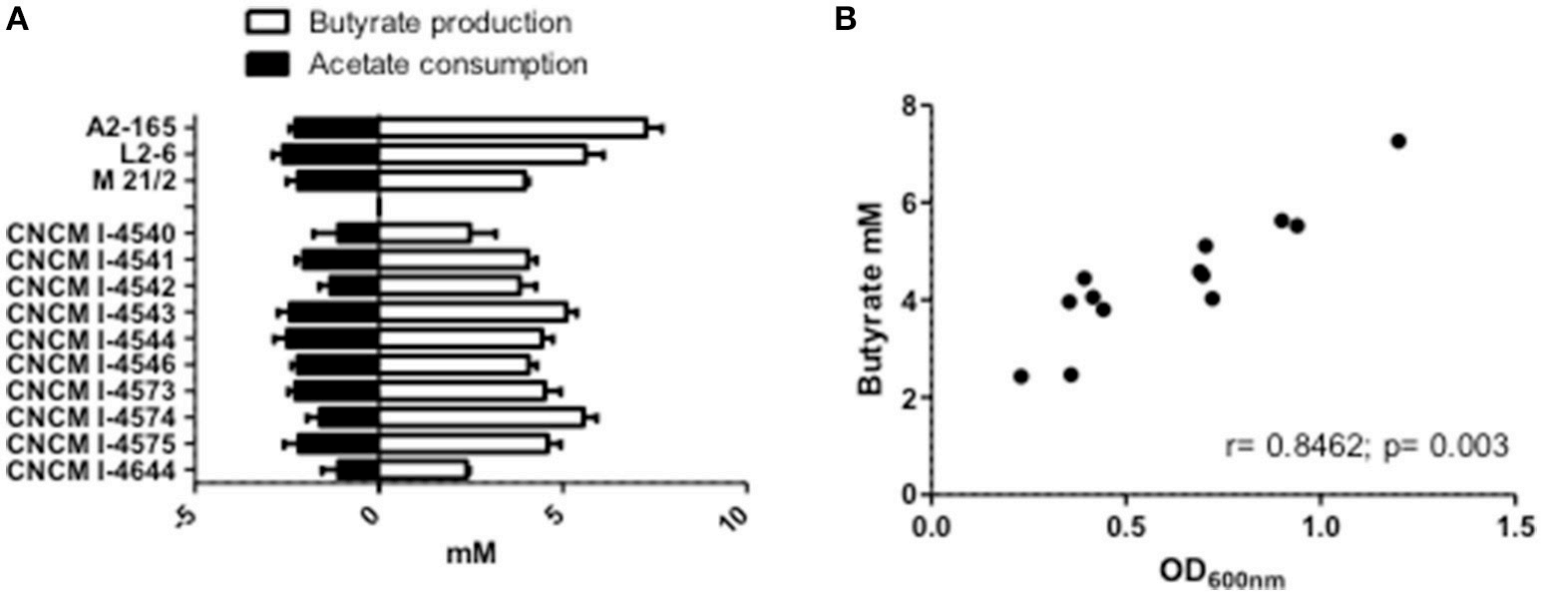
SCFA metabolism of *F. prausnitzii* strains *in vitro*. **(A)** Acetate and butyrate concentrations after 20 h growth of strain in YBHI supplemented medium. The concentration of control media was subtracted for each measurement have been done at least in triplicate. **(B)** Correlation between OD_600nm_ and butyrate production.

It is now well-establish that *F. prausnitzii* is an acetate-consumer and butyrate-producer species (Duncan et al., 2002; Lopez-Siles et al., 2012). Here, we report that in pure cultures, our new isolated strains are also able to produce butyrate and this production is significantly and positively correlated to their growth (OD_600nm_; *r* = 0.8462; *p* = 0.003; Figures 5A,B). It is interesting to highlight that the production level of butyrate was not linked to a particular phylogroup (Phylogroup I 3.91 mM ± 0.43 and Phylogroup II 4.89 mM ± 0.62). Moreover, all strains could metabolize acetate present in the culture medium at around the same level (Figure 5A). This consumption was not directly correlated to bacterial growth (*r* = −0.3132, *p* = 0.2975) and tended to be more correlated to butyrate production (*r* = −0.544, *p* = 0.0546). This observation is in agreement with the literature which describes that most of the carbon present in the butyrate produced (around 85%) is derived from external acetate, with only 15% provided directly from glucose (FEEDAP, 2012).

*F. prausnitzii* can also produce a few amount of D-lactate (FEEDAP, 2012). Indeed, among our strain collection, no L-lactate was detected and only few amounts of D-lactate were detected (1.09 mM ± 0.15 and 1.07 mM ± 0.39 phylogroup I and II respectively; data not shown). This production, not correlated with phylogroup affiliation, was correlated to the OD_600nm_(*r* = 0.6209, *p* = 0.0235). Bacterial D-Lactate production can be viewed as harmful since accumulation of this metabolite into the blood may be neurotoxic and leads to acidosis (Mack, 2004). In particular, humans with short bowel syndrome (in which small intestine has been surgically removed), the D/L fecal lactate ratio seems to be the most relevant index with a higher D-encephalopathy risk (Mayeur et al., 2013). However, in healthy adults, there is no lactate detectable in fecal samples, because lactobacilli (main producer of D-lactate) are minor groups in microbiota and lactate is degraded by other major bacterial groups (36, He, 2008 #41). This observation also suggested that the weak production of D-lactate by *F. prausnitzii* strains, major component of the microbiota, could not have metabolic deleterious impact on the host.

All strains were unable to growth in the presence of mucin as the only carbon source in a defined medium (data not shown). This data agrees with previous results where no evidence of fermentation of porcine gastric mucin by *F. prausnitzii* was detected (Lopez-Siles et al., 2012). Nevertheless, SCFA concentrations and OD_600nm_ measures taken after 2 days of incubation showed the ability of the different strains to survive but metabolically inactive as it could be deduced by the absence of butyrate in the supernatants of the cultures and the almost minimal OD_600nm_ recorded (data no shown). A decrease in butyrate production due to non-optimal growth conditions have been already reported for *F. prausnitzii* A2-165 strain (Lopez-Siles et al., 2012). This characteristic pointed out the intrinsic growth requirements of this species which, in addition to be an EOS, needs strain specific nutritional environment and has the ability to switch between substrates derived from the diet or the host (Lopez-Siles et al., 2017).

### Lytic activities

Gelatin is a heterogeneous mixture of water-soluble protein that is usually used in microbiological procedures to detect the presence of proteolytic activities. None of the strains were able to degrade gelatin in the conditions recommended by the API gallery supplier (data not shown). However, when the strains were inoculated in the gallery in a defined medium instead of API suspension medium, they were able to degrade partially this compound after 3 days of incubation. This fact suggests that the strains are able to hydrolyze gelatin although, maybe due to the growth limitations present in this culture media, the existence of this compound is not enough to allow the metabolic development of this activity in the strains.

The presence of hemolytic activity was tested using blood agar plates. None of the strains showed hemolytic activity under the conditions tested. In contrast, all the strains reveal a DNAse activity in green methyl-DNA medium (data not shown). Furthermore, the presence of a magnesium dependent DNase activity has been previously reported in at least three of five strains already sequenced [A2-165 (gi:257439194), SL3/3 (gi:295105207), and L2/6 (gi:295102777)].

The presence of these extracellular activities is often linked to a virulence status in some bacterial species such as *Enterococcus* spp. (Eaton and Gasson, 2001). However, these factors also contribute to the survival of microorganisms in the mammalian gut being characteristic of several members of the natural microbiota (Sanders et al., 2010). This can be the case of *Faecalibacterium* isolates, which are extremely well-adapted to the gut environment (Lopez-Siles et al., 2012).

### Antibacterial activities

We investigated antibacterial properties of *F. prausnitzii* supernatants, using the bacteriocin activity assay. We did not reveal any antibacterial effect on several anaerobic and aerobic bacterial species under the conditions tested. This fact is a desirable characteristic of a strain to be considered as a probiotic candidate.

### Ability to stimulate the immune response

**Figure 6:**
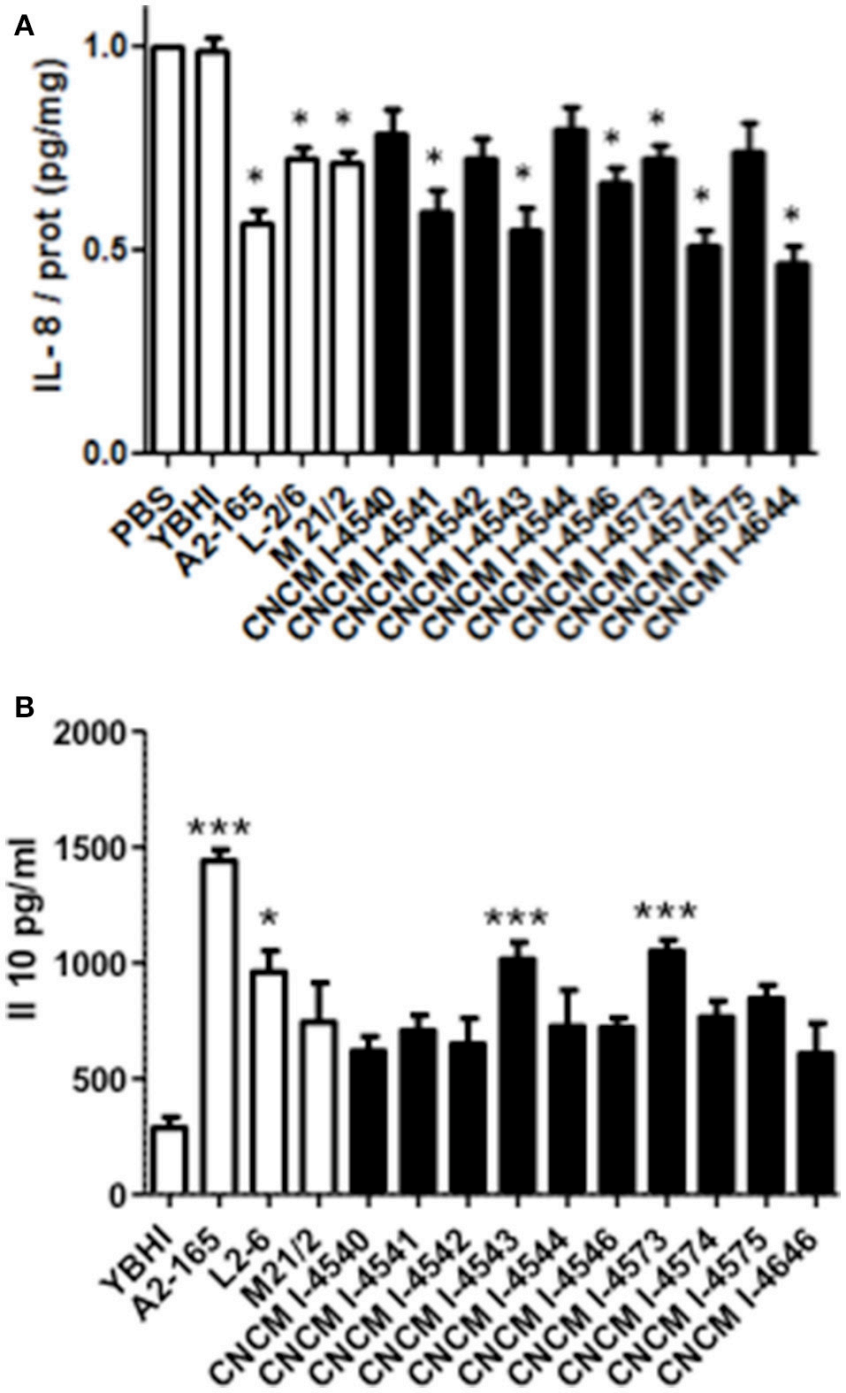
Immuno-modulation capacities of *F. prausnitzii* strains *in vitro*. **(A)** IL-8 production in HT-29 TNF-α stimulated cells. Experiments have been done at least in triplicate. Results are expressed as IL-8/ protein (pg/mg) and have been normalized using as reference value the IL-8 produced after the co-incubation with PBS as a negative control. **(B)** IL-10 production in peripheral blood mononuclear cells. Experiments have been done at least in triplicate. Results are expressed as IL-10 concentration (pg/mL). Significant differences from the control (YBHI) was specified as: ^*^p < 0.05 and ^***^p < 0.001.

The reference strain *F. prausnitzii* A2-165 is well-known for its immuno-modulatory properties and more specifically for its anti-inflammatory effects both *in vitro* and *in vivo* in different murine models of colitis (Sokol et al., 2008; Martin et al., 2014). To determine whether the newly isolated *F. prausnitzii* strains are able to modulate the immune response, we tested *in vitro* the immuno-modulatory properties of the supernatants from all the isolates in two different cellular models: HT-29 and PBMC. The first one is based on the capacity to block IL-8 production (a pro-inflammatory cytokine) induced by TNF-α stimulation in HT-29 epithelial cells and the second is based on the stimulation of PBMC cells and the measure of the anti-inflammatory cytokine IL-10. As shown in Figure 6A, all the strains tend to decrease IL-8 concentrations. However, this decrease was not equivalent in all the strains and does not correlate either with growth ratio (*r* = −0.2857, *p* = 0.344) or butyrate production (*r* = −0.3357, *p* = 0.2869).

For the PBMC assay, although all the strains tend to increase the production of IL-10 cytokine, only four strains (two controls and two new isolates from this study) were able to induce statistically significant increase production of this cytokine (Figure 6B) The two most performing strains (A2-165 and 4543) belong to the phylogroup II, group B. Notably, the IL-10 production was correlated with both growth ratio (*r* = 0.6813, *p* = 0.0103) and butyrate production (*r* = −0.6923, *p* = 0.0126). This different phenotype may suggest the presence of different molecule(s) responsible of the anti-inflammatory effects *in vitro*. The anti-inflammatory properties of butyrate have been already reported in the literature (Fusunyan et al., 1999; Kamitani et al., 1999) and its ability to block IL-8 production under the conditions tested in this study were confirmed *in vitro* in similar concentrations to those founds in *F. prausnitzii* supernatants (data not shown). However, its role remains controversial as its effects seems to be dose- and time-dependent as well as depended on the cellular model used (Martin et al., 2013). For instance, regarding cells from intestinal origin, butyrate has been found to decrease the secretion of IL-8 in Caco-2 and HIPEC cells and, in contrast to this study, to enhance IL-8 production in HT-29 and HT-29 MTX cells (Bocker et al., 2003).

However, several authors have found different candidate molecules/structures responsible for *F. prausnitzii* anti-inflammatory effects. MAM protein, found in *F. prausnitzii* supernatant, has been found to block NF-κB activation and the production of the pro-inflammatory cytokine IL-8 (Quevrain et al., 2016). *F. prausnitzii* is also able to produce bioactive anti-inflammatory molecules such as shikimic and salicylic acids (Miquel et al., 2015b). Besides, Rossi and co-workers showed the ability of *F. prausnitzii* strain HTF-F and its extracellular polymeric matrix to develop immunomodulatory effects through the TLR2 dependent modulation of IL-12 and IL-10 cytokine production in human monocyte-derived dendritic cells (Rossi et al., 2015) and *F. prausnitzii* has been found to be a strong inducer of regulatory T cells secreting IL-10 (Sarrabayrouse et al., 2014). All these results point out the complex anti-inflammatory mechanisms underlying this species.

### Adhesion to epithelial cells *in vitro*

**Figure 7:**
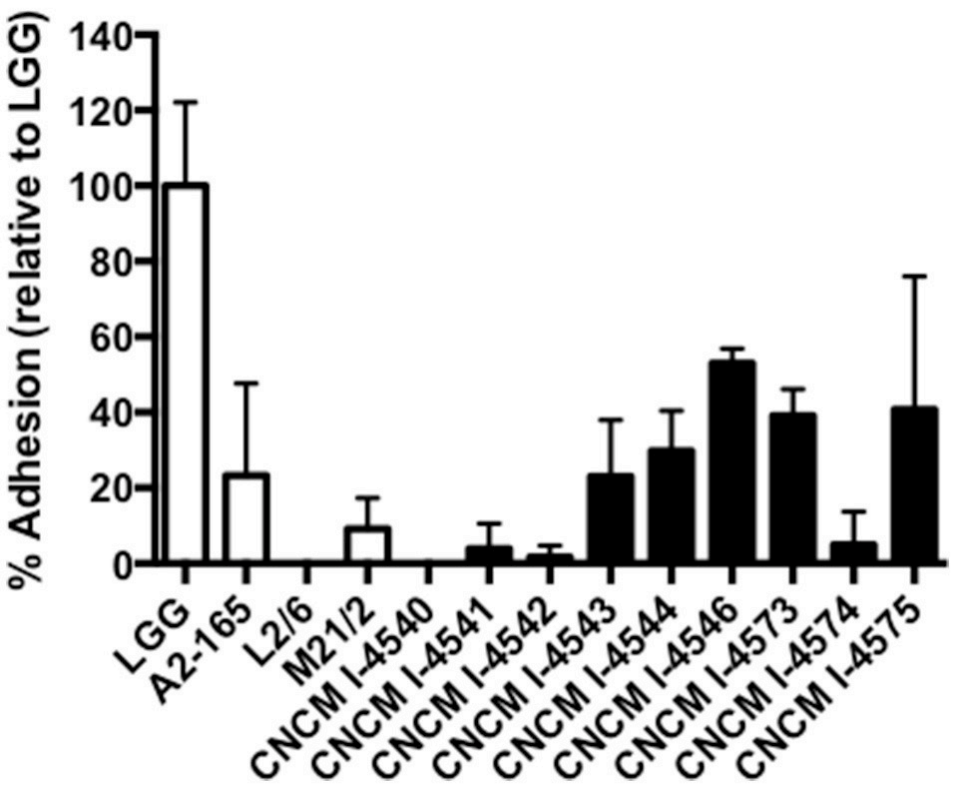
Adhesion to mucin of *F. prausnitzii* strains. Experiments have been done in triplicate. The adhesion values have been normalized using *Lactobacillus rhamnosus* GG (LGG) a positive control know by their good adhesion properties to mucin (50). Results are presented by the mean and the standard deviation.

In parallel, we also sought for the adhesion capacities of the new *F. prausnitzii* isolates to the intestinal epithelial cells HT-29 and mucin. All the tested strains were not able to adhere to HT-29 cells *in vitro* (data not shown) in anaerobic conditions. Regarding mucin, some of the strains were able to adhere to this compound after 3 h of incubation in the anaerobic chamber (Figure 7), Even if our conditions were not representative of physiological conditions (death of our eukaryotic cells), this result gives ecological clues about the processes of colonization of the gastro-intestinal tract by *F. prausnitzii*. In fact, this species is a late but major commensal colonizer of the gut which implantation requires a likely copro-cooperation maybe for the establishment of a trophic chain (Wrzosek et al., 2013).

## Concluding remarks

The development of new probiotic products containing human isolated strains with beneficial properties for the host requires the development of new techniques in order to: (i) isolate strains belonging to the major groups of the intestinal microbiota, (ii) determinate their safe status and (iii) infer in their potential beneficial effects. This study meets these entire three requests. Work with anaerobic and more precisely EOS bacteria are a prerequisite to succeed in the isolation of representative strains that can impact on intestinal homeostasis. For this reason, in this study, we have used a new procedure to isolate EOS strains from feces that has enabled us to build a collection of *F. prausnitzii* strains. The lack of knowledge about this species prompts us to further analyze their genetic diversity by comparing the new isolates with those already available in the databases. This has allowed us to point out the high diversity of our collection ranged on two different phylogroups with different clusters. *F. prausnitzii* strain genomes should be established or/and a metabolic comparison of several strains in the same culture conditions whether the phylogroups belong to genomovars or genomospecies.

Regarding safety concerns, this study is the first step toward a better understanding of *F. prausnitzii* properties. Up to date, little was known about *F. prausnitzii* resistance to antibiotics, lytic activities or adhesion properties. Here, we have shown for the first time the profile of all these characteristics in a collection of human *Faecalibacterium* strains. A positive remark is that all the strains were not antibacterial producers, not hemolytic and weak producer of D-lactate. Furthermore, although some of the strains were able to adhere to mucin, this trait can be considered as factor favoring durable implantation and a highly effective probiotic (Miquel et al., 2015a). However, further analyses are required to better determine the presence of acquired or natural resistances as well as to distinguish between the pathogenic or adaptative nature of some of the properties detected such as the presence of DNase activity.

Finally, the anti-inflammatory properties of all the strains have been analyzed. There is a well-known correlation between *F. prausnitzii* dysbiosis and a large set of human diseases such as IBD and IBS (Miquel et al., 2013). Recent studies using *F. prausnitzii* strains in *in vivo* models provide arguments concerning its beneficial effect on the host (Sokol et al., 2008; Wrzosek et al., 2013; Martin et al., 2014). The presence of the anti-inflammatory properties of these strains also opens the possibility to test them in murine models in order to further determine their beneficial effects before testing them in human clinical trials.

## Author contributions

RM, SM, JC, HS, LGBH, MT, and PL participate in the design of the project. RM, SM, JC, HS, OB, VA, LGBH, MT, and PL designed the experiments. RM, SM, LB, CB, VR, SH, and FC performed the experiments and analysis. RM and SM draft the manuscript. VR, CB, FC, JC, HS, LGBH, MT, and PL revised the manuscript critically. All the authors have read and approved the last version of the manuscript.

### Conflict of interest statement

PL and HS are co-founders of the start-up NextBiotiX aiming to use next-generation probiotics to fight and to prevent IBD. The other authors declare that the research was conducted in the absence of any commercial or financial relationships that could be construed as a potential conflict of interest.
